# The R.A.M.C. War Memorial

**Published:** 1919-05-17

**Authors:** 


### The Hospital

The Workers' Newspaper of Administrative Medicine and Institutional Life, Administration, National Insurance and Health.

No. 1719, Vol. LXVI. SATURDAY, MAY 17, 1919. PRICE TWOPENCE

### THE R.A.M.C. WAR MEMORIAL.

This is a memorial to the officers and men, 17,338 officers and 179,711 other ranks, who have served with the Royal Army Medical Corps throughout the war. Its first object will be to erect a memorial to the officers and men of all ranks who have fallen in the Great War. They number 560 officers and 4,091 others. A powerful and representative Committee, with Lieut.-Gen. Sir Alfred Keogh, G.C.B., at its head, has been formed, with representatives of the Regular, Territorial, Special Reserve and Temporary Commissioned Officers as members. The proposal is to erect a permanent memorial or monument in London, and it is hoped to place replicas in Edinburgh and Dublin. A fund will be formed to enable grants in aid to be given to the families of officers, non-commissioned officers and men of all branches of the R.A.M.C. who have fallen, or have been disabled in the war, and others who, owing to the exigencies of military service, are known to be in necessitous circumstances. H.R.H. Field-Marshal the Duke of Connaught, K.G., has consented to be the honorary chairman of the Committee. It is further hoped that money may be forthcoming to provide scholarships and memorial prizes for research work, which will be open to officers and men of the Corps.

These objects will commend themselves to all sections of His Majesty's subjects, and especially to members of the medical profession. The first point we wish to emphasise strongly is that money, and much of it, must be forthcoming to enable all the objects mentioned to be accomplished in the fullest and amplest possible manner. So far as the medical profession is concerned the public must grasp that the war, accompanied as it has been by the most patriotic, constant, and unselfish giving of themselves to the service of the nation throughout its duration, has meant loss, often serious loss, to a large number of practitioners. There are few indeed who have survived the war without loss, and fewer still who have made incomes sufficient to do more than pay their way during its course. We are anxious that the public shall apprehend these facts, at their true value, when considering the part they will take in the R.A.M.C. memorial.

The Memorial Committee, as we have pointed out, consists mainly of members of the profession. There is no doubt that both consultants and practitioners will do their best to support this war memorial to the officers and men. Our privilege to-day is, however, to deal especially with the part of members of the general public who, among all the claims on the nation, should feel that at any rate this one should appeal especially to each man and woman, who has the mind and will nobly to fulfil his or her duty to all, to whom we owe our freedom and the complete defeat of the enemy. Are there any amongst our fellow-countrymen who, with a clear conscience, can pass this memorial by? Let us briefly consider some facts of the position.

In all wars previous to the great war of 1914-19 disease destroyed armies in the past to an infinitely greater extent than any other cause. In the present war, in a general sense, the Expeditionary Force was the healthiest community in the world. It was mainly composed of young men of selected physique, well-fed and clothed, who led a healthy life in the open air under such sanitary conditions as were possible. The limitations of this possibility resulted from military necessities, such as overcrowding, huts, tents, barns, cellars, dug-outs, and billets. There was further the exhausting effect of exposure to bad weather, want of sleep, and neglect of personal cleanliness during the long periods of duty in the trenches. These unavoidable and irremediable exceptions to health conditions would have been quite sufficient to produce the diseases which destroyed armies in the past, but in the present war the long-hoped-for impossible, the miraculous, the inconceivable, materialised, and scientists have triumphed, and enteric fever and dysentery, fell diseases which had previously devastated armies in every part of the world, have been eliminated. Enteric is now a negligible factor and dysentery occurs only under exceptional circumstances. The public should recognise that the most successful Generals, after all, have been thos[illegible] Generals of Science in association with the Royal Army Medical Corps who have won such a decisiv[illegible] victory over the ubiquitous and elusive bacillus [illegible] dysentery, and the prevailing diseases in this wa[illegible] fortunately have been the simpler and milder types. The skill and thoroughness with which the battle with disease and the enforcement of preventive measures have been pursued, and their success, must ever redound to the glory and credit of the R.A.M.C. and those responsible. In previous wars typhoid fever frequently killed far more than the bullets of the enemy, whilst thousands of soldiers died of disease before they ever saw the opposing troops.

The present writer has been privileged to visit the fields of war, seeing for himself the wonderful work which the members of the R.A.M.C. of all ranks have accomplished, the dangers they have had to face, and the courage, devotion, and skill which has everywhere prevailed. Our fighting men have made a point of testifying that the dangers incurred by the officers and men of the R.A.M.C. and non-fighting ranks have equalled, if they have not surpassed, those of the actual combatants. At one time we were losing fifty men a month out of every 1,000 whose duty it was to attend the wounded on the field of battle and to bring them out from No Man's Land to the advance and dressing stations. We had the privilege of sharing these risks and making ourselves acquainted with their terrible nature. It is grateful to know that a way was found practically to eliminate these dangers and stop the casualties which at one time amounted to 600 out of every 1,000 per annum engaged. We have not space to set forth in detail the wonderful work done, or the immense and great developments which have taken place in surgical treatment and in hosts of other ways through the experience gained in the present war. It is but the truth that nothing which the members of the civilian population can do to mark their admiration for the glorious work thus accomplished through the R.A.M.C. in the present war can possibly be in excess of its merits. Nor can it be too great to adequately express our Nation and Empire's thanks for the work done, or the pride and gratitude felt by people of all classes and both sexes throughout the British Empire.

